# The GABA_A_ Receptor Influences Pressure Overload-Induced Heart Failure by Modulating Macrophages in Mice

**DOI:** 10.3389/fimmu.2021.670153

**Published:** 2021-05-31

**Authors:** Jin Bu, Shiyuan Huang, Jue Wang, Tong Xia, Hui Liu, Ya You, Zhaohui Wang, Kun Liu

**Affiliations:** ^1^Department of Pediatrics, Union Hospital, Tongji Medical College, Huazhong University of Science and Technology, Wuhan, China; ^2^Department of Geriatrics, Union Hospital, Tongji Medical College, Huazhong University of Science and Technology, Wuhan, China; ^3^Department of Hematology, Tongji Hospital, Tongji Medical College, Huazhong University of Science and Technology, Wuhan, China; ^4^Institution of Cardiology, Union Hospital, Tongji Medical College, Huazhong University of Science and Technology, Wuhan, China

**Keywords:** GABA_A_ receptor, amphiregulin, macrophage, monocyte, pressure-overload hypertrophy

## Abstract

**Background:**

Myocardial macrophages have key roles in cardiac remodeling and dysfunction. The gamma-aminobutyric acid subtype A (GABA_A_) receptor was recently found to be distributed in macrophages, allowing regulation of inflammatory responses to various diseases. This study aimed to clarify the role of GABA_A_ receptor-mediated macrophage responses in pressure overload-induced heart failure.

**Methods and Results:**

C57BL/6J mice underwent transverse aortic constriction for pressure-overload hypertrophy (POH) and were intraperitoneally treated with a specific GABA_A_ receptor agonist (topiramate) or antagonist (bicuculline). Echocardiography, histology, and flow cytometry were performed to evaluate the causes and effects of myocardial hypertrophy and fibrosis. Activation of the GABA_A_ receptor by topiramate reduced ejection fraction and fractional shortening, enlarged the end-diastolic and end-systolic left ventricular internal diameter, aggravated myocardial hypertrophy and fibrosis, and accelerated heart failure in response to pressure overload. Mechanistically, topiramate increased the number of Ly6C^low^ macrophages in the heart during POH and circulating Ly6C^high^ classic monocyte infiltration in late-phase POH. Further, topiramate drove Ly6C^low^ macrophages toward MHCII^high^ macrophage polarization. As a result, Ly6C^low^ macrophages activated the amphiregulin-induced AKT/mTOR signaling pathway, and Ly6C^low^MHCII^high^ macrophage polarization increased expression levels of osteopontin and TGF-β, which led to myocardial hypertrophy and fibrosis. Conversely, GABA_A_ receptor blockage with bicuculline reversed these effects.

**Conclusions:**

Control of the GABA_A_ receptor activity in monocytes/macrophages plays an important role in myocardial hypertrophy and fibrosis after POH. Blockade of the GABA_A_ receptor has the potential to improve pressure overload-induced heart failure.

## Introduction

Heart failure (HF) is a leading cause of morbidity and mortality and has a poor prognosis (1). Chronic pressure overload, as seen in persistent hypertension or aortic stenosis, is a major risk factor for HF (2). The pressure-overload heart experiences the transitions from compensated hypertrophic remodeling with preserved contractile function to eccentric hypertrophy with contractile dysfunction (3). Notwithstanding the fact that current drugs capable of inhibiting neuroendocrine signaling can promote hypertrophy regression, HF progression and its poor prognosis remain unavoidable. Recent clinical evidence indicates that the progression from HFpEF to HFrEF has a worse prognosis than HFrEF and president HFpEF that is not accompanied by ejection fraction (EF) declines during follow-up (4). This underscores the need to unravel the mechanism of compensation to decompensation HF transition.

Mechanical pressure-overload hypertrophy (POH) results in HF progression from EF preservation to EF reduction with myocardial hypertrophy and fibrosis. Recently, It was shown that macrophages acted as key innate immune cells with important roles in the initiation and development of cardiac remodeling and dysfunction in mice with POH (5, 6). Macrophages can be constitutively present within the myocardium like kupffer cells in the liver and derived from blood monocytes as well (7, 8). Macrophages of different origins exert distinct effects on either early or late phases of POH. The proliferation of cardiac resident Ly6C^low^ macrophages as a mechanism of self-renewal occurs within early-phase POH, and infiltrating macrophages arise from circulating classic Ly6C^high^ monocytes in late-phase POH (9). Macrophages are sensitive to their surroundings and alter their physiology in response to environmental factors (10). A previous study showed that infiltration of microRNA-155–expressing macrophages in response to pressure overload promoted cardiac inflammation, hypertrophy, and failure (11). By contrast, depletion of macrophages attenuated left ventricle (LV) remodeling and dysfunction in POH mice (12).

Gamma-aminobutyric acid subtype A (GABA_A_) receptors are the principal neurotransmitter receptors of the central nervous system(CNS), and they are also found to be distributed in immune cells, such as neutrophils, monocytes, and macrophages (13). Recently, we published a study showing that activation or inhibition of the GABA_A_ receptor exerted a pronounced effect on post-infarction ventricular remodeling by modulating monocyte/macrophage subsets (14). The GABA_A_ receptor has also been shown to affect macrophage recruitment and activity in tumor microenvironments and stress-induced colon inflammation (15, 16). Thus, the GABA_A_ receptor can influence pathological processes in various diseases *via* the modulation of macrophages. However, it remains unclear whether macrophage modulation affects pressure overload-induced cardiac remodeling and dysfunction, which have increasingly been associated with macrophage-mediated inflammation in POH.

Here, we sought to address the role of the GABA_A_ receptor in pressure overload-induced cardiac remodeling and dysfunction. In a transverse aortic constriction (TAC) model used to assess POH, we treated mice with a specific GABA_A_ receptor agonist (topiramate) or antagonist (bicuculline). We demonstrated that activation of the GABA_A_ receptor by topiramate accelerated HF. Conversely, blockade of the GABA_A_ receptor by bicuculline protected POH mice against LV enlargement and EF reduction. The data indicated that GABA_A_ receptor modulation would become a new strategy in the treatment of patients with pressure overload-induced HF.

## Materials and Methods

### Animals and Treatments

Six- to 8-week-old male C57BL/6J mice, weighing 22 to 24 g, were housed at room temperature under a 12-h light/dark cycle. Food and water were provided freely. The surgical and experimental procedures were approved by Animal Care and Use Committee of Tongji Medical College, Huazhong University of Science and Technology.

After 1 week of adaptive feeding, mice were assigned into either a sham surgery group or a transverse aortic constriction (TAC) group. Subsequently, mice in the TAC group were randomly assigned to one of the following treatments: (1) Sodium chloride(NaCl)-treated group (vehicle), NaCl at 8 ml/kg; (2) topiramate-treated group, topiramate at 35 mg/kg; (3) bicuculline-treated group, bicuculline at 2 mg/kg (provided by Sigma-Aldrich, USA). The four groups were named as follows: sham, vehicle, topiramate-treated TAC group, and bicuculline-treated TAC group. Starting 6 h after TAC surgery, mice were intraperitoneally (i.p.) injected once a day consecutively for 3 weeks. At the end of the study, the hearts were harvested and weighed to compare the heart weight/body weight (HW/BW, mg/g). Then, LV tissues were collected for further experiments.

### Neutralizing Antibody Administration

Mice were intraperitoneally administered five micrograms of affinity-purified anti-mouse-AREG polyclonal antibody (AF989, R&D systems) or IgG immediately before TAC and every 2 days after TAC.

### Surgical Protocol

TAC model was performed as described previously (17). In brief, mice were anaesthetized by isoflurane and artificially ventilated at a rate of 120 strokes per minute using a rodent ventilator with a mixture of O2 and air (1:2 vol/vol). Then, the aorta was constricted between the innominate and left common carotid arteries by placing a 5-0 nylon suture against a blunted 26-gauge needle. Blood pressure gradient across ligation site was confirmed by echography. Mice in sham group were subjected to identical operation without reducing aorta diameter to 26-gauge.

### Echocardiography

A Vevo1100 high-resolution *in vivo* imaging system (Fujifilm Visualsionics) was used for echocardiographic analysis. Mice were anesthetized with isoflurane. The LV B-mode and M-mode images were captured from parasternal short-axis or long-axis views at the level of the papillary muscles (18). The LV end-diastolic diameter (LVIDd) and LV end-systolic diameter (LVIDs) were measured from M-mode tracings of parasternal short-axis views at the mid and apical levels. The ejection fraction (EF) was calculated as EF (%) = 100 × [(LV Vol;d − LV Vol;s)/LV Vol;d]. LV Mass was calculated as LV mass (mg) = 1.053 × [(LVID;d + LVPW;d + IVS;d)^3^ − LVID;d^3^]. The fractional shortening (FS) was calculated as FS(%) = 100 × [(LVID;d − LVID;s)/LVID;d].

### Histological Analysis and Immunofluorescence

The cross-sectional area of cardiomyocytes (CMs) was assessed on the basis of wheat germ agglutinin (WGA) staining. For that, 5 µm thick paraffin-embedded sections from mice hearts were deparaffinized, rinsed in PBS and subsequently stained with Alexa Fluor 488-conjugated WGA according to the manufacturer-provided protocol. For the quantification of the cross-sectional area, slides were imaged by a fluorescence microscope and only CMs that were aligned transversely were considered. Cell area was calculated in more than 200 cells.

To determine collagen deposition, paraffin sections (5 µm thick) of hearts were stained with Masson’s trichrome as described previously (19). Perivascular fibrosis was calculated as the perivascular collagen area relative to the vessel area, and interstitial fibrosis was the percentage of the Azan’s trichrome-stained area per total area of cardiac tissue. The blinded measurements were made by two independent observers using ImagePro Plus 6.0 (Media Cybernetics, USA). To further detect collagen content, sections were stained in Sirius Red solution as described previously (20). Type I collagen fibrosis was determined as the ratio of red/yellow-stained area to total collagen-stained area.

For co-immunostaining of F4/80 and amphiregulin (Areg), antigen retrieval was performed by using boiling citrate buffered saline for 2 min after de-waxing and washing the sections in PBS. The heart slides were blocked for 1 h with 10% normal donkey serum. The tissue sections were incubated overnight with anti-Areg (A12680, ABclonal) and anti-F4/80 (Ab6640, Abcam) antibodies. Next day, the sections were washed and incubated for 2 h with Alexa-Fluor-488–conjugated anti-goat or Alexa-Fluor-594–conjugated anti-rat secondary antibodies (Invitrogen). DAPI was used to stain the cell nuclei. The stained samples were viewed using a confocal laser-scanning microscope.

### Flow Cytometry

To determine monocytes and their subsets or neutrophils, peripheral blood was collected in 50 mM EDTA (Sigma-Aldrich, USA) as anticoagulant at the day of sacrifice. Spleens and femurs were excised after vascular perfusion with sterile PBS. Spleens were triturated in PBS with the end of a 1-ml syringe, and filtered through 40 μm nylon mesh. Flushed bone marrow was also strained through the nylon mesh. Then, the cell suspension was centrifuged and resuspended to get single cell suspension. The total viable cell numbers were determined using neubauer chamber (BD Bioscience, USA) with Trypan blue(Sigma-Aldrich, USA). Cell suspension were incubated with antibodies against CD45 (eBioscience, USA), Ly-6G (eBioscience, USA), Ly-6C (eBioscience, USA), and CD11b (eBioscience, USA) for 30 min at 4°C, and washed twice for flow cytometry (FCM) analysis.

To analyze macrophages and their subsets, heart macrophages were collected as described previously (14). The right atria, left atria, and atrioventricular valves were removed after the whole heart tissue was excised. Then, LV was cut into small pieces in PBS and digested with collagenase B. The cell suspension was filtered and resuspended to get single cell suspension. After total viable cells were calculated, they were conjugated with the following antibodies for the identification defined as Ly-6C^high^ and Ly-6C^low^ macrophages according to Ly-6C (eBioscience, USA), CD45 (eBioscience, USA), F4/80 (eBioscience, USA), and CD11b (eBioscience, USA). After incubated with these antibodies for 30 min at 4°C, the cells were washed twice. To analyze the subsets of Ly-6C^low^ macrophages, cardiac single cell suspension was got according to the above. Cell suspension was incubated with antibodies against CD45 (eBioscience, USA), Ly-6C (eBioscience, USA), F4/80 (eBioscience, USA), MHCII (eBioscience, USA), and CD11b (eBioscience, USA) for 30 min at 4°C, and washed twice for FCM analysis. Similarly, to analyze the expression of Ki-67 protein, CD45 (eBioscience, USA), Ly-6C (eBioscience, USA), F4/80 (eBioscience, USA), Ki-67 (eBioscience, USA), and CD11b (eBioscience, USA) were incubated for FCM analysis.

#### EdU Incorporation Assay

To assess DNA synthesis in cardiac macrophages, 200 µg of EdU (5-ethynyl-2′-deoxyuridine) was injected into mice (i.p). Two hours after the injection, the heart was excised, and cell suspension was dispersed from the heart tissue and analyzed for incorporated EdU using FCM.

### Western Blot Analysis

Western blot was performed as described previously (21). LV tissue was homogenized in radioimmunoprecipitation buffer containing 1% protease inhibitor cocktail, and the concentration of the supernatant was measured by means of BCA. After the target proteins were loaded onto an SDS polyacrylamide gel, proteins were transferred from the gel to a PVDF membrane. Subsequently, the membranes were incubated overnight with Areg, mTOR (A2445, ABclonal), AKT (A18120, ABclonal), P-AKT (AP0655, ABclonal), or P-mTOR (AP0094, ABclonal) antibody after being blocked for 1 h. After washed in TBST, the membranes were incubated with HRP-linked secondary antibody for 60 min at room temperature. The protein bands were detected using an enhanced chemiluminescence agent (ECL reagents) and analyzed.

### RNA Extraction and Quantitative Real-Time PCR (RT-PCR) Analysis

The total RNA of the LV were isolated using TRIzol^®^Reagent, and then, cDNA was synthesized using Prime Script™ RT Master Mix(Takara, Japan). The internal control was glyceraldehyde-3-phosphate dehydrogenase (GAPDH). All reactions were performed in a 10-μl volume-containing 1 μl cDNA, 5 μl SYBR-Green reaction mix, 0.2 μl sense primer, 0.2 μl anti-sense primer (both from TSINGKE), 0.4 μl ROX, and 3.2 μl ddH2O twice. All reactions were performed in a Applied Biosystems. Relative changes were determined using the 2^−ΔΔCt^ method. The primer sequences were as follows:

GAPDH-F CAGTGGCAAAGTGGAGATTGTTG GAPDH-R TCGCTCCTGGAAGATGGTGAT
Ccl2-F CAGGTCCCTGTCATGCTTCT  Ccl2-R CCCATTCCTTCTTGGGGTCA
Cxcl 12-F CCTTCAGATTGTTGCACGGC  Cxcl 12-R TTACAAAGCGCCAGAGCAGA
osteopontin-F TTGCTTGGGTTTGCAGTCTTC osteopontin-R TATAGGATCTGGGTGCAGGCT
TGF-B1-F AGGGCTACCATGCCAACTTC  TGF-B1-R CCACGTAGTAGACGATGGGC

### Statistical Analysis

All values were expressed as the mean ± SEM and multiple group comparisons were analyzed by one-way analysis of variance (ANOVA), followed by Bonferroni’s test. The survival rate was analyzed by Kaplan–Meier survival analysis and compared by the log-rank test. A value of *P* < 0.05 was regarded to be statistically significant. The statistical analysis was performed with SPSS 17.0 and GraphPad Prism 7.0.

## Results

### Activation or Blockade of the GABA_A_ Receptor Aggravates or Attenuates Pressure Overload-Induced Cardiac Dysfunction

**Figure 1:**
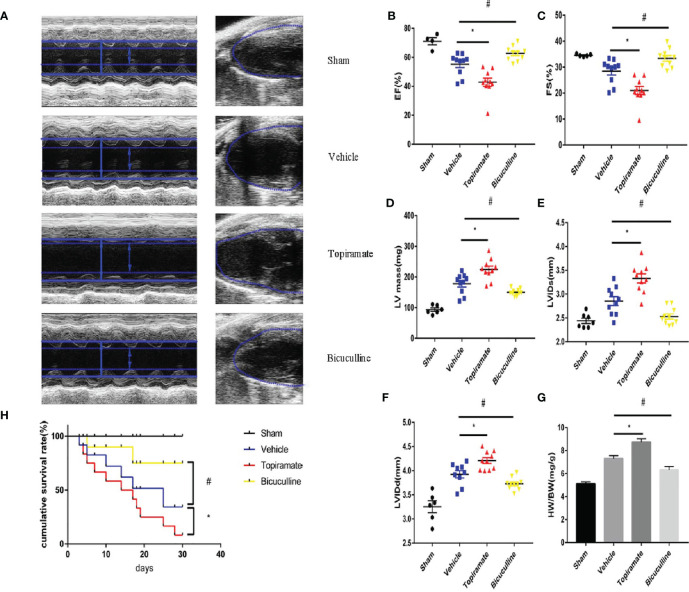
Activation or blockade of the GABA_A_ receptor aggravates or attenuates cardiac dysfunction in pressure-overload hypertrophy mice. Mice underwent sham or transverse aortic constriction (TAC) operation; mice in the TAC group were treated with intraperitoneal injections once a day of NaCl, topiramate or bicuculline for 3 weeks. **(A)** Representative left ventricular echocardiographic recordings at day 28 post-TAC. Short-axis M-mode (left) and long-axis B-mode (right). Arrows and lines marked (left ventricle internal dimension) LVID in systole (dashed) and diastole (firm). **(B)** Ejection fraction (%EF), **(C)** fractional shortening (%FS), **(D)** left-ventricular mass (LV mass), **(E)** left ventricle internal dimension in systole (LVIDs), and **(F)** in diastole (LVIDd) in TAC- and sham-operated mice at day 28 post-TAC. **(G)** Heart weight/body weight (HW/BW) in indicated groups at day 28 post-TAC. Data showed the mean ± SEM of %FS, %EF, LV mass, LVIDd, LVIDs and HW/BW for each experimental group, by one-way ANOVA with Bonferroni’s multiple comparison test. **(H)** Compared with vehicle, the survival rate increased in bicuculline-treated TAC group during 30 days follow-up post-TAC (n = 20 in each group). For topiramate treatment, *P < 0.05 *vs.* vehicle. For bicuculline treatment, ^#^P < 0.05 *vs.* vehicle. NaCl-treated TAC mice (for simplicity, vehicle). (n = 4–6 in sham group, n = 10 in TAC-operated group).

To assess the effect of the GABA_A_ receptor on TAC-induced cardiac function, echocardiography was performed in topiramate- or bicuculline-treated TAC mice. Four weeks after TAC, the transition from concentric to dilated hypertrophy in the LV was accelerated by topiramate, while the development of HF was attenuated by bicuculline. At day 28 post-TAC, topiramate-treated TAC mice showed significantly reduced cardiac systolic function, expressed as fractional shortening (FS) or ejection fraction (EF) in comparison to vehicle. By contrast, cardiac systolic function in bicuculline-treated TAC mice was significantly improved compared with vehicle (**Figures 1B, C** and **Supplementary Table 1**). Four weeks after pressure overload, cardiac hypertrophy parameters including end-diastolic and end-systolic left ventricular internal diameter (LVIDd and LVIDs), LV mass, and heart-to-body weight, were significantly aggravated by topiramate, and attenuated by bicuculline (**Figures 1A, D–G** and **Supplementary Table 1**). The survival rate was observed up to 30 days post-TAC, which was significantly higher in bicuculline-treated than that in topiramate-treated TAC mice and vehicle (**Figure 1H**).

### Activation or Blockade of the GABA_A_ Receptor Promotes or Alleviates Pressure Overload–Induced Myocardial Remodeling

**Figure 2:**
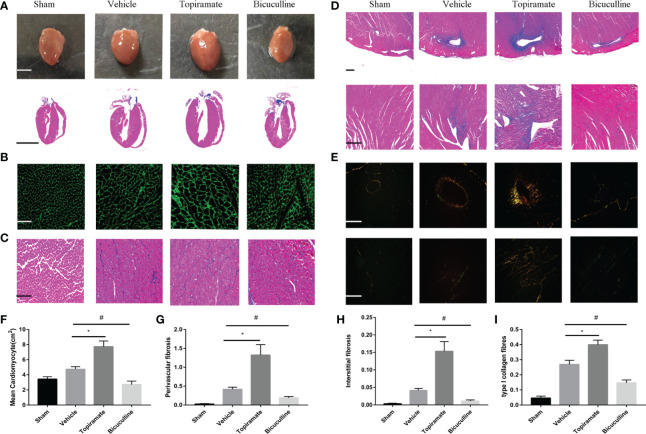
Activation or blockade of the GABA_A_ receptor promotes or alleviates myocardial remodeling in pressure-overload hypertrophy mice. **(A)** Representative visible images of hearts (top) and Masson trichrome staining of heart vertical section (bottom) at day 28 post-TAC. Scale bar = 2.5 mm. **(B, C)** Cardiomyocytes by wheat germ agglutinin (WGA) staining **(B)**, and Masson trichrome staining **(C)** at day 28 post-TAC. scale bar = 50 μm. **(D, E)** Cardiac fibrosis by Masson trichrome staining **(D)**, and Sirius Red staining **(E)** at day 28 post-TAC. (top, perivascular fibrosis; bottom, interstitial fibrosis). **(F–I)** Quantified analysis of cardiomyocytes size **(F)**, the perivascular collagen area relative to the vessel area **(G)**, the percentage of interstitial fibrosis area in each picture **(H)**, and the ratio of type I collagen fibrosis to total collagen-stained area **(I)**. Sections were analyzed with Image-Pro Plus 6.3 software. Data show the mean ± SEM, by one-way ANOVA with Bonferroni’s multiple comparison test. For topiramate treatment, *P < 0.05 *vs.* vehicle. For bicuculline treatment, ^#^P < 0.05 *vs.* vehicle. (each group, n = 6–10).

Consistent with findings in cardiac function, topiramate-treated TAC mice showed more severe myocardial hypertrophy, and interstitial or perivascular fibrosis, compared with vehicle. As shown in **Figures 2A–C, F**, cross-sectional area of individual cardiomyocyte measured respectively by WGA or Masson trichrome staining increased as well as visible heart enlargement did in topiramate-treated TAC mice, compared with vehicle. Bicuculline-treated TAC mice had a variance effect. In addition, Masson trichrome and Sirius Red staining showed that areas of interstitial or perivascular fibrosis and I type collagen ratio after TAC were significantly increased by topiramate treatment (**Figures 2D, E, G–I**). However, cardiac fibrosis was significantly less prominent in mice treated with bicuculline after TAC than in vehicle. Furthermore, the GABA_A_ receptor agonist or antagonist had no effect on the structure of the aorta, mesenteric arteries, and kidneys (**Supplementary 1**).

### Activation or Blockade of the GABA_A_ Receptor Selectively Increases or Reduces Accumulation of Ly6C^low^ Macrophages in the Heart

**Figure 3:**
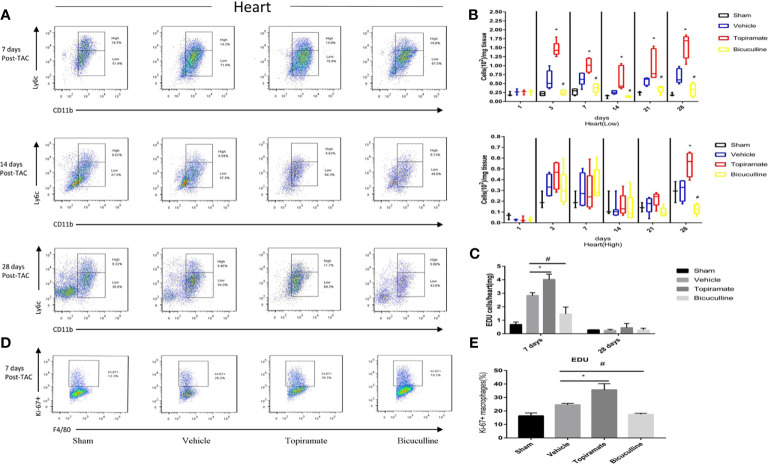
Activation or blockade of the GABA_A_ receptor selectively increases or reduces the number of Ly6C^low^ macrophages in the hearts of pressure-overload hypertrophy mice. Macrophage (CD45^+^F4/80^+^CD11b^+^) subpopulations were respectively defined as Ly6C^high^ or Ly6C^low^ macrophages according to Ly-6C expression levels. **(A)** Representative images of Ly6C^high^ and Ly6C^low^ macrophages at days 7, 14, and 28 post-TAC. The representative images of 1, 3, and 21 days after TAC were shown in supplementary materials. **(B)** The number of Ly6C^low^ macrophages or Ly6C^high^ macrophages (per mg heart tissue) among the total number of live cells isolated from hearts at the indicated time points after TAC. **(C)** The number of CD45^+^CD11b^+^F4/80^+^EdU^+^cell (per mg heart tissue) among the total number of live cells isolated from hearts at the indicated time points after TAC. **(D)** Representative images of Ki-67^+^ expression in Ly6C^low^ macrophages at day 7 post-TAC. **(E)** The percentage of Ki-67^+^ expression in Ly6C^low^ macrophages. Data show the mean ± SEM, by one-way ANOVA with Bonferroni’s multiple comparison test. For topiramate treatment, *P < 0.05 *vs.* vehicle. For bicuculline treatment, ^#^P < 0.05 *vs.* vehicle. (n = 3 mice for sham group, n = 6–8 mice for all other groups).

To assess the effect of the GABA_A_ receptor on macrophage polarization after TAC, we analyzed macrophage subpopulations in the heart by FCM at days 1, 3, 7, 14, 21, and 28 post-TAC. We used a Ly-6C marker to identify two subsets of macrophages with distinct functions, namely Ly6C^high^ and Ly6C^low^ macrophages. As shown in **Figures 3A, B**, compared with vehicle, topiramate resulted in the expansion of CD45^+^CD11b^+^F4/80^+^Ly6C^low^ macrophages (hereafter referred to as Ly6C^low^ macrophages) in the myocardium at days 3, 7, 14, 21, and 28 post-TAC, whereas bicuculline treatment exhibited opposite effects. Mice treated with either topiramate or bicuculline after TAC did not have significantly altered CD45^+^CD11b^+^F4/80^+^Ly6C^high^ macrophage populations (hereafter referred to as Ly6C^high^ macrophages), compared with vehicle at days 1, 3, 7, 14, and 21 post-TAC (**Figures 3A, B** and **Supplementary 2**). However, topiramate treatment markedly induced the expansion of Ly6C^high^ macrophages at day 28 post-TAC (late-phase POH) compared to vehicle (**Figures 3A, B****)**.

Recent studies have shown that cardiac Ly6C^low^ macrophages were independent of monocyte infiltration in early-phase POH. We hypothesized that activation of the GABA_A_ receptor increased levels of Ly6C^low^ macrophages by stimulating local proliferation in early-phase POH. Indeed, we found that activation of the GABA_A_ receptor led to the local proliferation of macrophages, as demonstrated by 5-ethynyl-2′-deoxyuridine (EdU) staining at day 7, but not day 28 post-TAC (**Figure 3C** and **Supplementary 2**). In parallel, activation of the GABA_A_ receptor significantly induced the expression of the Ki-67 protein, a nuclear proliferation marker, in Ly6C^low^ macrophages at day 7 post-TAC (**Figures 3D, E****)**. Collectively, these results suggest that activation of the GABA_A_ receptor increases the number of Ly6C^low^ macrophages by stimulating local proliferation of macrophages in early-phase POH.

### Activation or Blockade of the GABA_A_ Receptor Promotes or Reduces Monocyte Recruitment in Late-Phase POH

**Figure 4:**
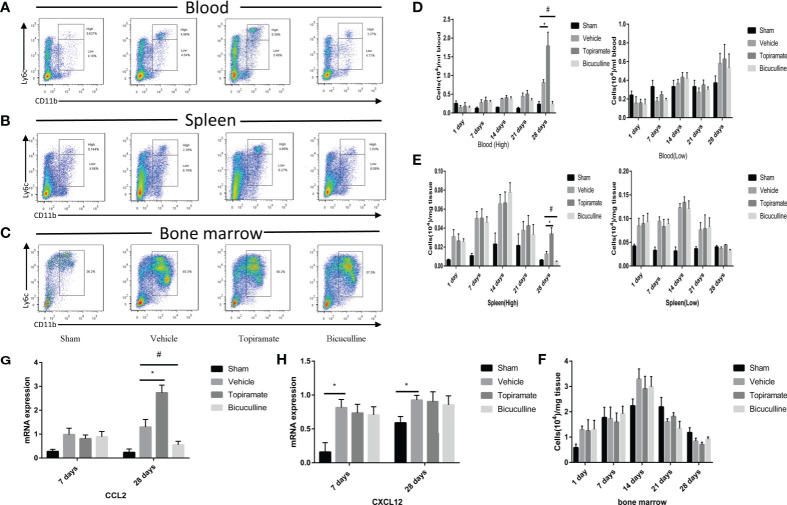
Activation or blockade of the GABA_A_ receptor promotes or reduces monocyte recruitment in pressure-overload hypertrophy mice. **(A, B)** Representative images for flow cytometric analysis of CD45^+^Ly6G^-^CD11b^+^Ly6C^high^ and CD45^+^Ly6G^-^CD11b^+^Ly6C^low^ monocytes in the blood **(A)**, and spleen **(B)** at day 28 post-TAC. **(C)** Representative images for flow cytometric analysis of monocytes (CD45^+^Ly6G^-^CD11b^+^Ly6C^+^) in bone marrow at day 28 post-TAC. The representative images of other time points are shown in supplementary materials. **(D)** The number of Ly6C^high^ or Ly6C^low^ monocytes (per ml blood) among the total number of live cells isolated from blood at the indicated time points. **(E)** The number of Ly6C^high^ or Ly6C^low^ monocytes (per mg spleen) among the total number of live cells isolated from spleen at the indicated time points. **(F)** The number of monocytes (per mg marrow bone) among the total number of live cells isolated from marrow bone at the indicated time points. **(G, H)** Expression of Ccl2 and Cxcl12 marker genes at days 7 and 28 post-TAC. Data show the mean ± SEM, by one-way ANOVA with Bonferroni’s multiple comparison test. For topiramate treatment, *P < 0.05 *vs.* vehicle. For bicuculline treatment, ^#^P < 0.05 *vs.* vehicle. (n = 3 mice for sham group, n = 6–8 mice for all other groups).

To further explore the effect of the GABA_A_ receptor on systemic innate immunity in mice after TAC, we analyzed the number of monocyte subsets (CD45^+^Ly6G^-^CD11b^+^Ly6C^high^ or CD45^+^Ly6G^-^CD11b^+^Ly6C^low^) in the peripheral blood and spleen, and monocytes (CD45^+^Ly6G^-^CD11b^+^Ly6C^+^) in marrow bone by FCM at days 1, 7, 14, 21, and 28. The results showed that the GABA_A_ receptor exerted no influence on monocytes in the peripheral blood, spleen, and marrow bone at days 1, 7, 14, and 21 post-TAC (**Figures 4D–F** and **Supplementary 3–5**). However, treatment with either topiramate or bicuculline markedly affected Ly6C^high^ monocyte counts in the peripheral blood and spleen not in marrow bone at day 28 post-TAC (late-phase POH) (**Figures 4A–F**). For example, topiramate treatment resulted in Ly6C^high^ monocyte counts in the peripheral blood and spleen to increase from 0.81 ± 0.07 × 10^4^ to 1.78 ± 0.37 × 10^4^, and 0.01 ± 0.01 × 10^4^ to 0.03 ± 0.01 × 10^4^, respectively. Furthermore, blood neutrophil counts were evaluated at days 7 and 28 post-TAC, revealing that counts in topiramate/bicuculline-treated TAC mice did not differ from those in vehicle (**Supplementary 6**).

To determine whether the accumulation of myocardial macrophages depends on the recruitment of monocytes from blood in late-phase POH, we measured chemokine expression in myocardial tissues at days 7 and 28 post-TAC. Intriguingly, chemokine C-C motif ligand-2 (CCL2) levels were elevated in topiramate-treated TAC mice versus vehicle at day 28, but not day 7 post-TAC (**Figure 4G**), whereas CCL2 levels in bicuculline-treated TAC mice were lower than those in vehicle. We also found that topiramate treatment increased the percentage of CCR2^+^ macrophages in the heart at day 28 post-TAC (**Supplementary 7**). However, in topiramate/bicuculline-treated TAC mice, chemokine C-X-C motif ligand-12 (Cxcl12) levels were unaltered at days 7 and 28 post-TAC, which aligned well with unaltered blood neutrophil densities (**Figure 4H** and **Supplementary 6**). These data demonstrate that activation of the GABA_A_ receptor induces circulating Ly6C^high^ classic monocyte infiltration in late-phase POH *via* CCL2/CCR2-dependent monocyte migration.

### GABA_A_ Receptor-Mediated Myocardial Hypertrophy Is Associated With Areg-Induced Akt/mTOR Signaling

**Figure 5:**
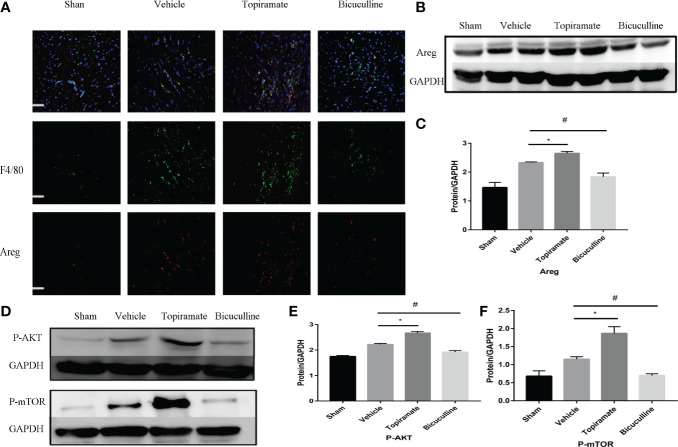
Areg expression and Akt/mTOR signaling are involved in the GABA_A_ receptor-mediated cardiac hypertrophy. **(A)** Representative micrographs showing immunofluorescent staining of Areg (red) and a macrophage marker, F4/80 (green) in a sample of heart tissue. Nuclei were counterstained with DAPI (blue). Scale bar = 50 µm. **(B)** Representative images of Areg protein at day 7 post-TAC in heart. **(D)** Representative images of P-Akt and P-mTOR protein at day 7 post-TAC in heart. **(C, E, F)** Quantitative analysis of Areg protein **(C)**, P-Akt protein **(E)**, and P-mTOR protein **(F)** at day 7 post-TAC in heart. GAPDH mAb (middle) was used as a loading control. Data are mean ± SEM, by one-way ANOVA with Bonferroni’s multiple comparison test. For topiramate treatment, *P < 0.05 *vs.* vehicle. For bicuculline treatment, ^#^P < 0.05 *vs.* vehicle. (each group, n = 6).

**Figure 6:**
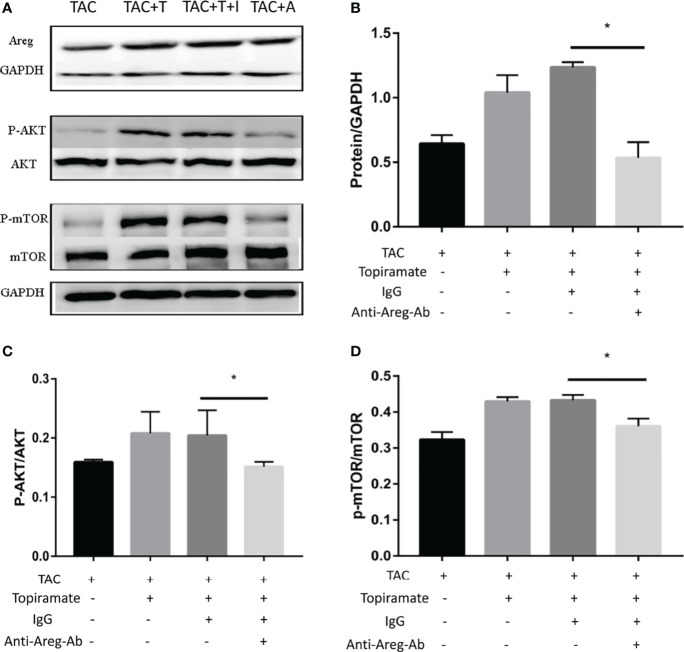
Blocking of Areg activity suppresses topiramate-mediated effect on Akt/mTOR signaling. **(A)** Representative images of Areg, P-Akt, and P-mTOR proteins at day 7 post-TAC in heart. T: topiramate; I: IgG; A: Anti-Areg Ab + IgG + topiramate. **(B–D)** Quantitative analysis of Areg protein **(B)**, P-Akt protein **(C)**, and P-mTOR protein **(D)** at day 7 post-TAC in heart. Data are mean ± SEM, by one-way ANOVA with Bonferroni’s multiple comparison test. *P < 0.05 *vs.* TAC + Topiramate+ IgG. (each group, n = 6).

To elucidate the mechanism by which the GABA_A_ receptor affects cardiac macrophages and thus, induces myocardial hypertrophy, we tested whether humoral factors produced from macrophages affected cardiomyocytes. Areg has recently been shown to induce cardiomyocyte hypertrophy (22). We found that topiramate increased the areas of regions that co-stained positive for Areg and F4/80 and upregulated Areg protein levels in the heart. Conversely, bicuculline treatment significantly reduced the area of co-stained regions and levels of Areg protein expression (**Figures 5A–C**). Furthermore, increased cardiomyocyte size in topiramate-treated mice was accompanied by increasing levels of phosphorylation of AKT and mTOR, while bicuculline reduced phosphorylation levels of both proteins relative to those which were observed in vehicle (**Figures 5D–F** and **Supplementary 8**). The capability of the GABA_A_ receptor activation to induce Akt and mTOR phosphorylation was blocked by a neutralizing antibody specific for Areg (**Figure 6**). These data suggest that Areg-induced Akt/mTOR signaling pathway may be essential for GABA_A_ receptor-mediated cardiac hypertrophy.

### The GABA_A_ Receptor Affects Fibrosis by Regulating MHCII^low^/MHCII^high^ Ratios in Subpopulations of Ly6C^low^ Macrophages

**Figure 7:**
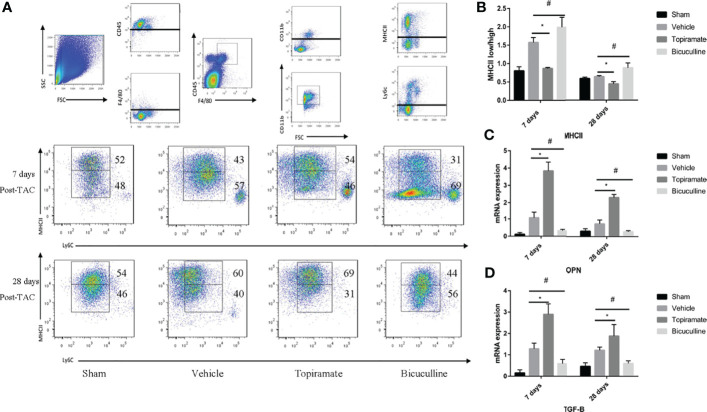
The GABA_A_ receptor affects fibrosis by regulating MHCII^low^/MHCII^high^ ratios in subpopulations of Ly6C^low^ macrophages. Ly6C^low^ macrophages could be further classified into two subpopulations based on MHC II proteins. **(A)** Representative images of MHC II ^high^ and MHC II ^low^ macrophages at days 7 and 28 post-TAC in heart. **(B)** Quantified analysis of the percentage of MHC II ^low^ and MHC II ^high^ macrophages at days 7 and 28 post-TAC. **(C, D)** Relative mRNA expression of OPN **(C)**, and TGF-B marker genes **(D)** at days 7 and 28 post-TAC in the heart. Data show the mean ± SEM, by one-way ANOVA with Bonferroni’s multiple comparison test. For topiramate treatment, *P < 0.05 *vs.* vehicle. For bicuculline treatment, ^#^P < 0.05 *vs.* vehicle. (each group, n = 6).

To understand how the GABA_A_ receptor affects fibrosis, we assessed the crosstalk that occurred between macrophages and fibroblasts. As reported, Ly6C^low^ macrophages can be further classified into two subpopulations based on expression levels of major histocompatibility complex (MHC) class II proteins. We found that topiramate treatment reduced MHCII^low^/MHCII^high^ ratios in subpopulations of Ly6C^low^ macrophages, and bicuculline increased the ratios of MHCII^low^/MHCII^high^ at days 7 and 28 post-TAC, compared with vehicle (**Figures 7A, B****)**. Furthermore, RT-PCR revealed that topiramate treatment increased osteopontin (OPN) and TGF-β transcript levels at days 7 and 28 post-TAC, whereas bicuculline treatment reduced their expression levels, compared with vehicle (**Figures 7C, D****)**. These data suggest that activation of the GABA_A_ receptor polarizes macrophages toward Ly6C^low^MHCII^high^ subpopulations after pressure overload in the heart.

## Discussion

In this study, we identified the role of GABA_A_ receptors in pressure overload-induced HF. Using the GABA_A_-specific receptor agonist or antagonist, we found that activation of the GABA_A_ receptor by topiramate resulted in cardiac dysfunction, as shown by worsened FS & EF, enlarged LVIDd & LVIDs, and aggravation of cardiac hypertrophy and fibrosis, in comparison to vehicle. In contrast, blockade of the GABA_A_ receptor by bicuculline reversed this effect in mice with POH, protecting them against cardiac dysfunction and hypertrophy. Mechanistically, topiramate stimulated the local proliferation of cardiac resident macrophages, which were identified as Ly6C^low^ macrophages during early-phase POH, and promoted bloodborne monocyte recruitment and macrophage polarization in late-phase POH. We also found that topiramate promoted the polarization of Ly6C^low^ macrophages labeled with MHCII^high^. As a consequence of Ly6C^low^ macrophage polarization by topiramate treatment, the Areg-induced Akt/mTOR signaling pathway was activated, which resulted in myocardial hypertrophy. With a decrease in the ratios of MHCII^low^/MHCII^high^ subpopulations of Ly6C^low^ macrophages, OPN and TGF-B levels increased, thereby contributing to fibrosis. As the antagonist, bicuculline exhibited the opposite effect.

TAC is a classic model that presents dynamic changes of pressure overload-induced hypertrophy and HF (23). In response to TAC, as it is commonly held, the compensatory cardiac hypertrophy occurs within the initial 2 weeks for the sake of preserved contractile function (24). In a period ranging from 2 to 4 weeks after TAC, decompensation and HF manifest (9). Here, we present findings indicating that macrophages are a potential pharmacological target against pressure overload-induced HF. Intriguingly, bicuculline, a GABA_A_ receptor antagonist that is clinically used as an analgesic, produced novel effect and emerged as a chemical candidate for the reversal of cardiac dysfunction due to pressure overload. It exerted a significant influence on myocardial hypertrophy as well as fibrosis, contrasted by topiramate. By affecting both the pathological changes underlying pressure overload-induced HF, the process of POH can be modulated. Therefore, during an initial or early phase, blockade of the GABA_A_ receptor made it possible to manipulate cardiac resident macrophages to achieve the goal of reversing POH.

Recently, increasing evidence has indicated that the GABA_A_ receptor is enriched in immune cells, where it exhibits anti- or pro-inflammatory effects. Previous findings showed that activation of the GABA_A_ receptor could potently inhibit post-infarction inflammation by acting upon bloodborne or cardiac resident macrophages (14). Moreover, Kim et al. suggested that GABAergic activation promoted macrophage antimicrobial responses against mycobacterial infection (25). However, activation of the GABA_A_ receptor A3 subunit has been shown to impair colon barrier function by upregulating macrophage activity in early-life stress mice (16). In comparison, inhibition of the GABA_A_ receptor reduced neuroinflammation in the hippocampus and CNS (26, 27). These diverse effects might be due to GABA_A_ receptor activation in different pathological situations.

In early-phase POH, GABA_A_ receptor activation or blockade did not affect neutrophils and monocytes, whereas it promoted or suppressed local proliferation of cardiac resident macrophages marked with Ly6C^low^ as shown in **Figures 3** and **4**. These results are in accordance with those of Liao X et al. who showed that cardiac residence in macrophages is a mechanism of self-renewal, which occurred within early-phase POH. Increasing evidences support that GABAergic drugs can cause CNS or myocardial pathologic changes through activation or blockade of the GABA_A_ receptor in macrophages independent of CNS (14, 26). A recent study shows that Ly6C^low^ macrophages can induce a hypertrophic response in cardiomyocytes for myocardial adaptive response to pressure overload (22). Evidence indicates that cardiac resident macrophages have tissue-repairing capacities after injuries (28). In mice with myocardial infarction (MI), proliferation of cardiac resident macrophages together with monocyte-derived Ly6C^low^ macrophages activated by topiramate attenuated ventricular remodeling by promoting neovascularization and fibrosis instead of infarcts. However, activation of the GABA_A_ receptor compelled cardiac resident macrophages to play a new role in ventricular remodeling in response to pressure overload. These players contribute to cardiac hypertrophy and fibrosis by producing effectors. For instance, Areg, a humoral factor specifically produced by Ly6C^low^ macrophages, induces a hypertrophic response and activates AKT/mTOR signaling which is proportional to rate of eccentric hypertrophy progression under pressure overload (29, 30).

Similar to monocyte-derived Ly6C^low^ macrophages, cardiac resident macrophages can also promote fibrosis. As reported recently, these Ly6C^low^ macrophages can be further classified into MHCII^high^ or MHCII^low^ macrophages based on MHCII expression levels (28). MHCII^low^ macrophages favor matrix breakdown, whereas MHCII^high^ macrophages display enrichment in a gene set that regulates fibrosis. The high ratios of MHCII^high^/MHCII^low^ macrophages may induce cardiac fibrosis, which leads to diastolic dysfunction in mice with hypertension or advanced age (31). Our data showed that activation of the GABA_A_ receptor increased the ratios of MHCII^high^/MHCII^low^. Due to the shift toward MHCII ^high^ macrophages, TGF-B or OPN expression was upregulated, thereby aggravating cardiac fibrosis.

In late-phase POH, which was accompanied by EF reduction and enlargement of LV, Ly6C^high^ monocytes infiltrated the myocardium and were derived into macrophages, which participated in myocardial injury and repair responses. Thus, macrophages acted similar to MI.

The mechanism of GABAergic drugs on cultured peritoneal macrophages and bone marrow-derived macrophages was ever investigated (13, 25). However, cardiac resident macrophages emerge in recent years with little knowledge of their nature *in vivo* or vitro and their subtypes (7, 32). Thus, it is imperative to gain more knowledge in future.

In conclusion, our study provides novel insights into the role of cardiac resident macrophages and GABA_A_ receptor in pressure overload-induced HF. The pros and cons of our results show that cardiac resident macrophages can probably act as key players in the progression from HFpEF to HFrEF. Thus, targeting the GABA_A_ receptor in cardiac resident macrophages will likely benefit the early treatment of HFpEF. And the antagonist or blocker emerges as a promising therapeutical chemical.

## Data Availability Statement

The original contributions presented in the study are included in the article/**Supplementary Material**. Further inquiries can be directed to the corresponding authors.

## Ethics Statement

The animal study was reviewed and approved by Animal Care and Use Committee of Tongji Medical College, Huazhong University of Science and Technology. Written informed consent was obtained from the owners for the participation of their animals in this study.

## Author Contributions

JB and ZW conceived the study. JB, YY, TX, HL, and JW performed the experiments and data analyses. KL and SH provided intellectual inputs. JB and KL wrote the manuscript. All authors edited and approved the manuscript. All authors contributed to the article and approved the submitted version.

## Funding

This work was supported by the National Natural Science Foundation of China (81901429 to SH and 82070514 to KL).

## Conflict of Interest

The authors declare that the research was conducted in the absence of any commercial or financial relationships that could be construed as a potential conflict of interest.
