# Corrigendum: The GABA_A_ Receptor Influences Pressure Overload-Induced Heart Failure by Modulating Macrophages in Mice

**DOI:** 10.3389/fimmu.2021.753404

**Published:** 2021-09-21

**Authors:** Jin Bu, Shiyuan Huang, Jue Wang, Tong Xia, Hui Liu, Ya You, Zhaohui Wang, Kun Liu

**Affiliations:** ^1^Department of Pediatrics, Union Hospital, Tongji Medical College, Huazhong University of Science and Technology, Wuhan, China; ^2^Department of Geriatrics, Union Hospital, Tongji Medical College, Huazhong University of Science and Technology, Wuhan, China; ^3^Department of Hematology, Tongji Hospital, Tongji Medical College, Huazhong University of Science and Technology, Wuhan, China; ^4^Institution of Cardiology, Union Hospital, Tongji Medical College, Huazhong University of Science and Technology, Wuhan, China

**Keywords:** GABA_A_ receptor, amphiregulin, macrophage, monocyte, pressure-overload hypertrophy

In the original article, there was a mistake in **Figure 3A** and **Supplement 3** as published**.** In the **Figure 3A**, the bicuculine group at day 28 post-TAC was inadvertently saved in the improper folders and attached to Sham group at days 14 and 28 post-TAC, so the representative images of Sham group at days 14 and 28 post-TAC were wrong and duplicated. In the **Supplement 3**, the representative image of Sham group at day 21 post-TAC was chosen by mistake. The corrected **Figure 3A** and **Supplement 3** appear below.

**Figure 3:**
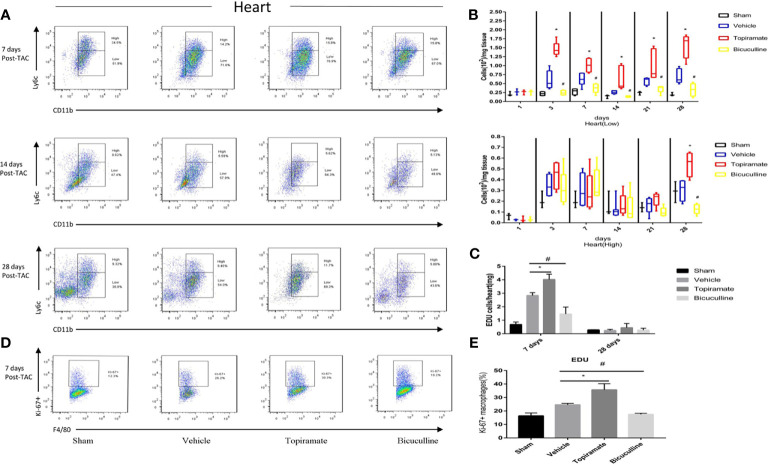
Activation or blockade of the GABA_A_ receptor selectively increases or reduces the number of Ly6C^low^ macrophages in the hearts of pressure-overload hypertrophy mice. Macrophage (CD45^+^F4/80^+^CD11b^+^) subpopulations were respectively defined as Ly6C^high^ or Ly6C^low^ macrophages according to Ly-6C expression levels. **(A)** Representative images of Ly6C^high^ and Ly6C^low^ macrophages at days 7, 14, and 28 post-TAC. The representative images of 1, 3, and 21 days after TAC were shown in supplementary materials. **(B)** The number of Ly6C^low^ macrophages or Ly6C^high^ macrophages (per mg heart tissue) among the total number of live cells isolated from hearts at the indicated time points after TAC. **(C)** The number of CD45^+^CD11b^+^F4/80^+^EdU^+^cell (per mg heart tissue) among the total number of live cells isolated from hearts at the indicated time points after TAC. **(D)** Representative images of Ki-67^+^ expression in Ly6C^low^ macrophages at day 7 post-TAC. **(E)** The percentage of Ki-67^+^ expression in Ly6C^low^ macrophages. Data show the mean ± SEM, by one-way ANOVA with Bonferroni’s multiple comparison test. For topiramate treatment, *P < 0.05 *vs*. vehicle. For bicuculline treatment, ^#^P < 0.05 *vs.* vehicle. (n = 3 mice for sham group, n = 6–8 mice for all other groups).

The authors apologize for this error and state that this does not change the scientific conclusions of the article in any way. The original article has been updated.

## Publisher’s Note

All claims expressed in this article are solely those of the authors and do not necessarily represent those of their affiliated organizations, or those of the publisher, the editors and the reviewers. Any product that may be evaluated in this article, or claim that may be made by its manufacturer, is not guaranteed or endorsed by the publisher.

## Supplementary Material

Supplement 3 Living cells (upper panel) were gated to determine the presence of CD45^+^Ly6G^-^CD11b^+^Ly6C^high^ and CD45^+^Ly6G^-^CD11b^+^Ly6C^low^ monocytes at day 21 post-TAC in the peripheral blood.

