# Supplementary material for: The GABAA Receptor Influences Pressure Overload-Induced Heart Failure by Modulating Macrophages in Mice
Source: Front Immunol. 2021 May 31;12:670153. doi: 10.3389/fimmu.2021.670153 (PMC8201502; doi:10.3389/fimmu.2021.670153)
Supplement: Supplementary file 1 [file DataSheet_1.docx]

SUPPLEMENTAL MATERIAL

|  | Sham | Vehicle | Topiramate | Bicuculline |
| --- | --- | --- | --- | --- |
| EF，% | 71.04±2.49 | 55.23±2.39 | 42.82±2.86* | 62.7±1.55# |
| FS，% | 34.55±0.21 | 28.47±1.48 | 21±1.56* | 33.37±1.11# |
| LVmass | 93.47±5.56 | 178±10.33 | 224.6±10.84* | 150.2±3.48# |
| LVID.d | 3.25 ± 0.12 | 3.93±0.07 | 4.21±0.06* | 3.72±0.04# |
| LVID.s | 2.44±0.06 | 2.85±0.09 | 3.33±0.09* | 2.52±0.05# |
| Peak Grad | 3.8±1.21 | 64.32±0.80 | 64.82±0.29 | 65.29±0.31 |
| Peak Vel | -938.8 ± 148.8 | -4007 ± 28.86 | -3936 ± 89.64 | -3948 ± 80.19 |

Table 1: Data are means ± SEM, by one-way ANOVA with Bonferroni’s multiple comparison test (n=4-6 in Sham group, n = 10 in TAC-operated group). For topiramate treatment, *P＜0.05 vs. vehicle. For bicuculline treatment, ^#^P＜0.05 vs. vehicle.


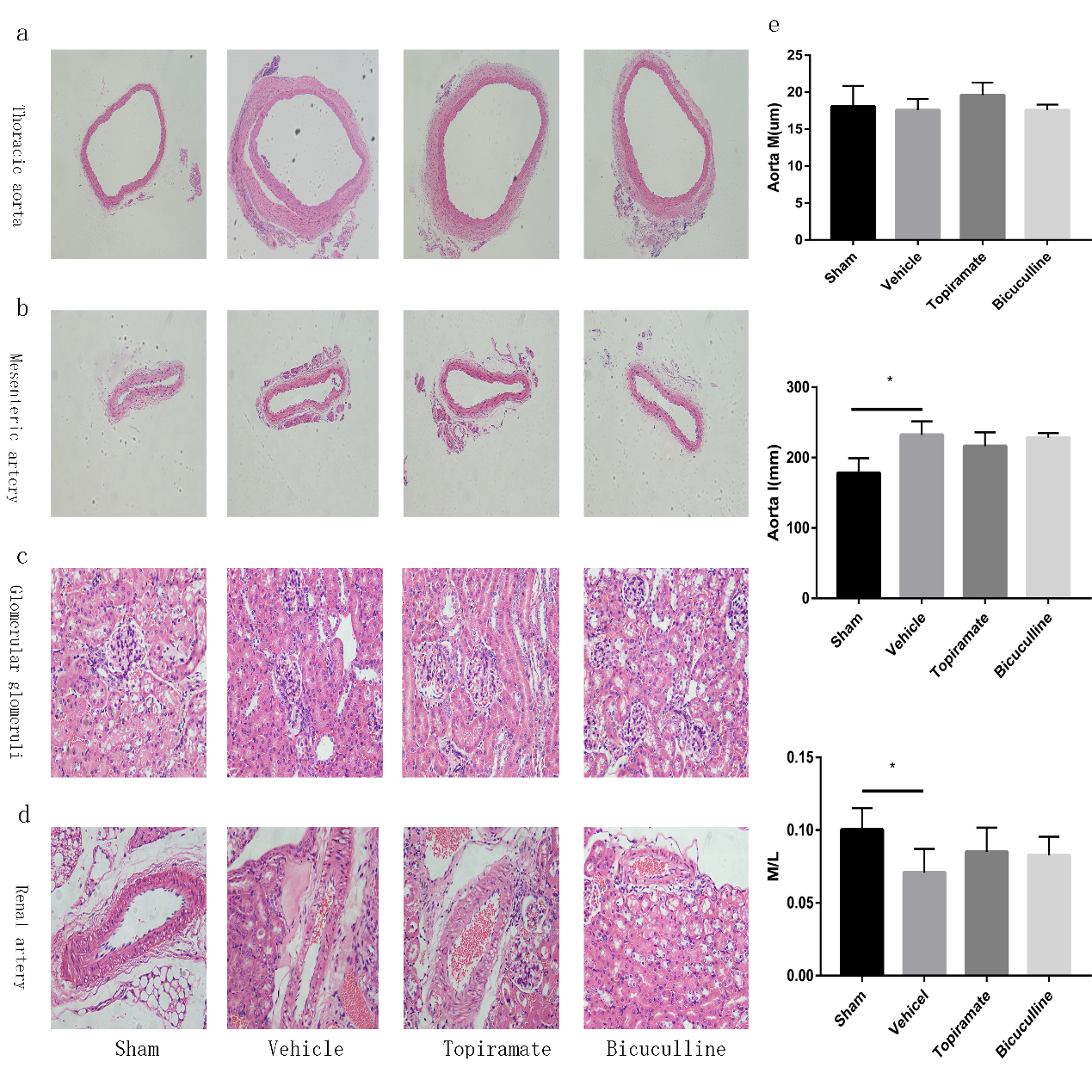


Supplement 1: Histology of aorta, mesenteric artery, and kidney. (A-D) Representative images at day 28 post-TAC. Thoracic aorta(a), Mesenteric artery(b), Glomerular glomeruli(c), and Renal artery(d). (e) Quantified analysis of thoracic aorta. aorta M (Media); aorta L(lumen); M/L (Media to Lumen ratio). Data are mean ± SEM. *P＜0.05 vs. vehicle.


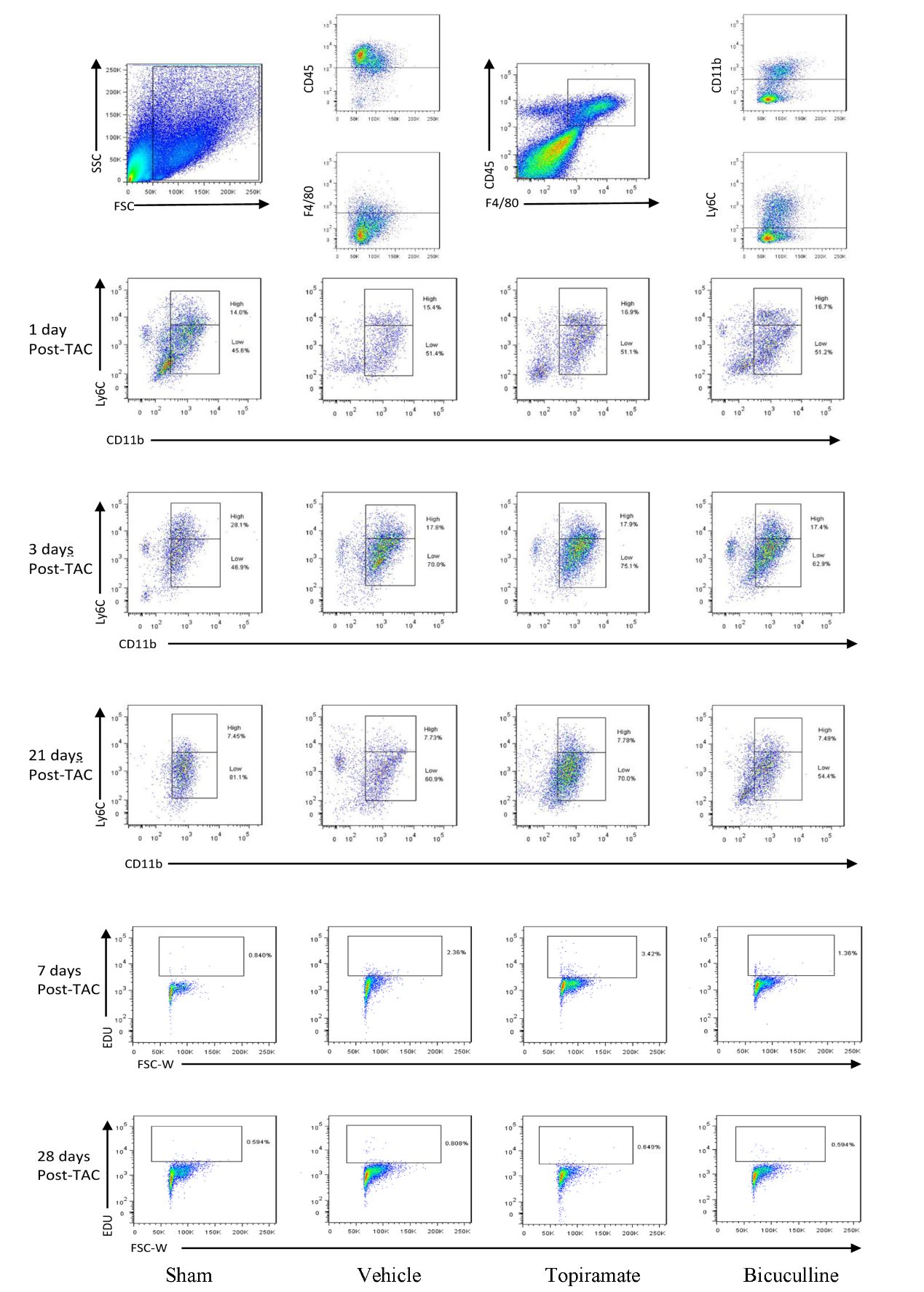


Supplement 2: (a) Living cells (upper panel) were gated to determine the presence of CD45^+^F4/80^+^CD11b^+^Ly6C^high^ and CD45^+^F4/80^+^CD11b^+^Ly6C^low^ macrophages at days 1, 3 and 21 post-TAC in heart. (b) Representative images of CD45^+^CD11b^+^F4/80^+^EdU^+^ cell fractions at days 7 and 28 post-TAC in heart.


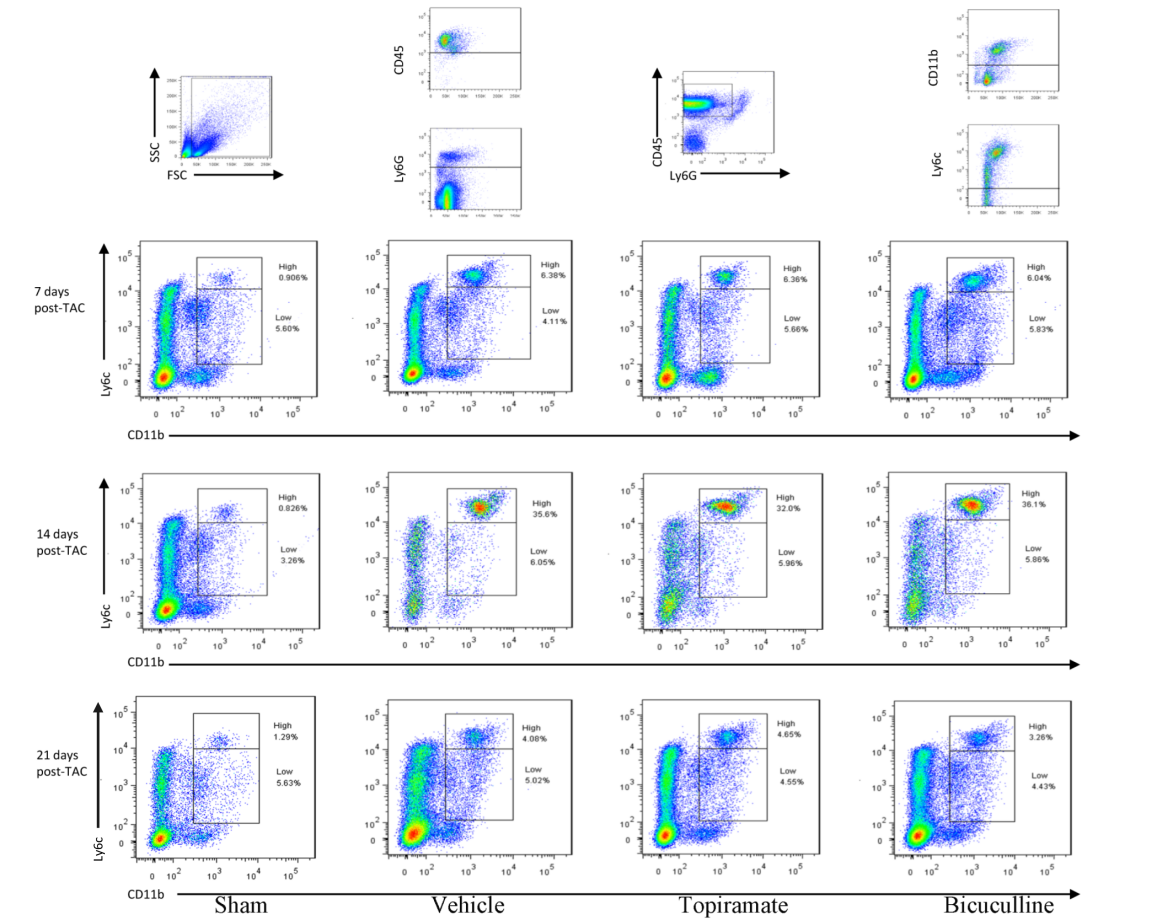


Supplement 3: Living cells (upper panel) were gated to determine the presence of CD45^+^Ly6G^-^CD11b^+^Ly6C^high^ and CD45^+^Ly6G^-^CD11b^+^Ly6C^low^ monocytes at days 7, 14 and 21 post-TAC in the peripheral blood.


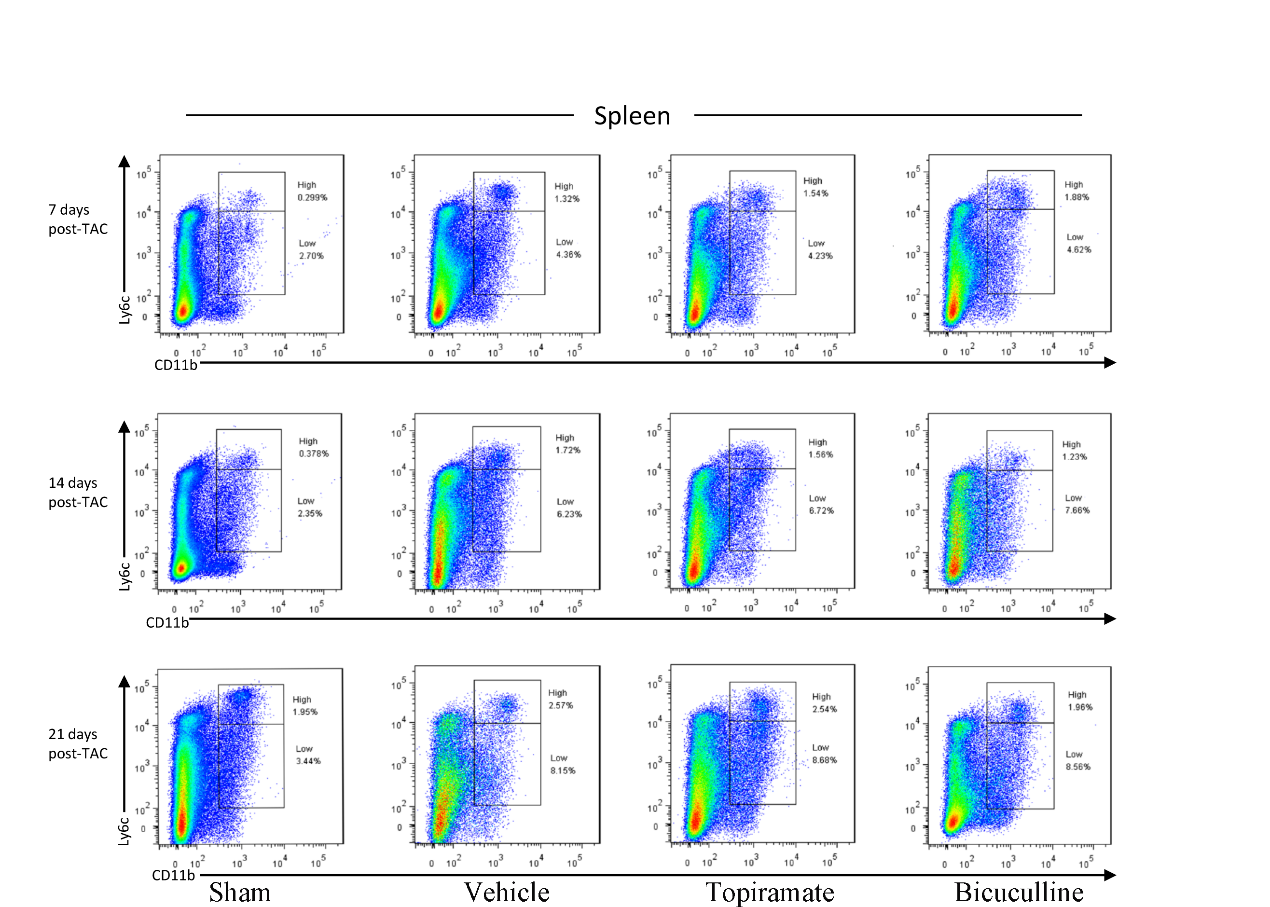


Supplement 4: Representative images for flow cytometric analysis of CD45^+^Ly6G^-^CD11b^+^Ly6C^high^ and CD45^+^Ly6G^-^CD11b^+^Ly6C^low^ monocytes at days 7, 14 and 21 post-TAC in the spleen.


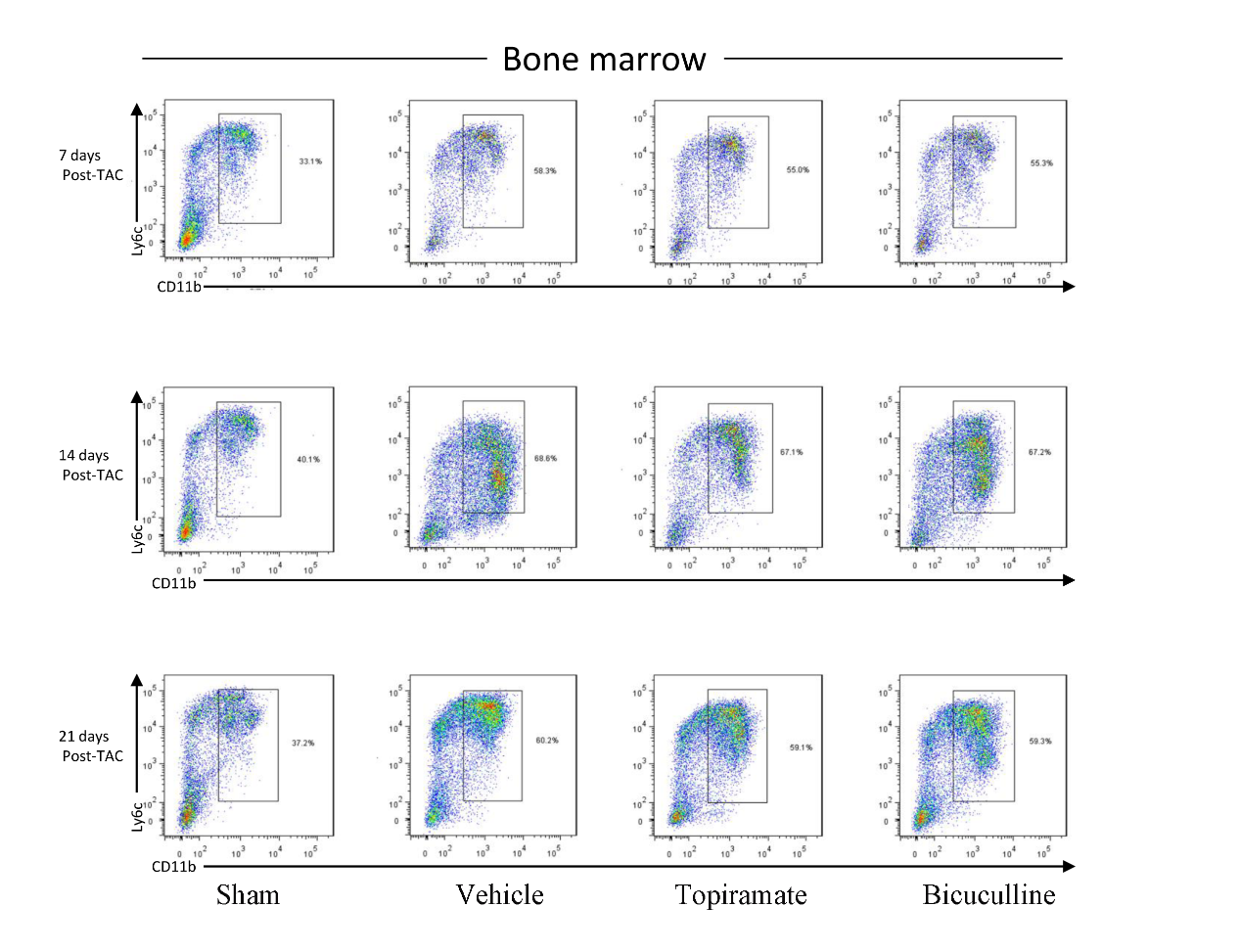


Supplement 5: Representative images for flow cytometric analysis of CD45^+^Ly6G^-^CD11b^+^Ly6C^+^ monocytes at days 7, 14 and 21 post-TAC in the bone marrow.


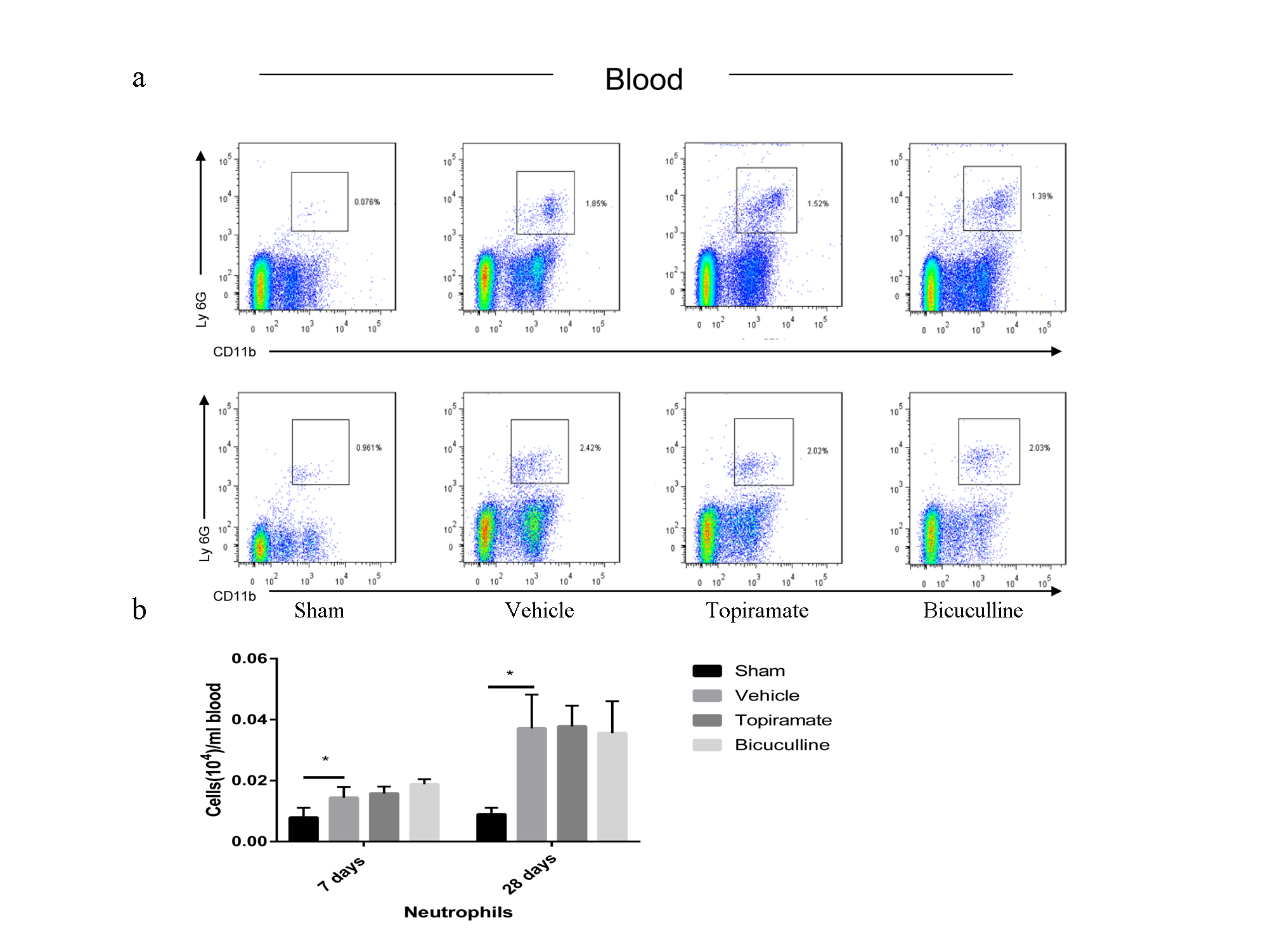


Supplement 6: (a) Representative images for flow cytometric analysis of CD45^+^Ly6G^+^CD11b^+^ neutrophils (lower left) and counted (lower right) at days 7and 28 post-TAC in the peripheral blood. (b) The number of neutrophils (per ml blood) among the total number of live cells isolated from blood at the indicated time points after TAC. Data are mean ± SEM, *P≤0.05 vs.vehicle.


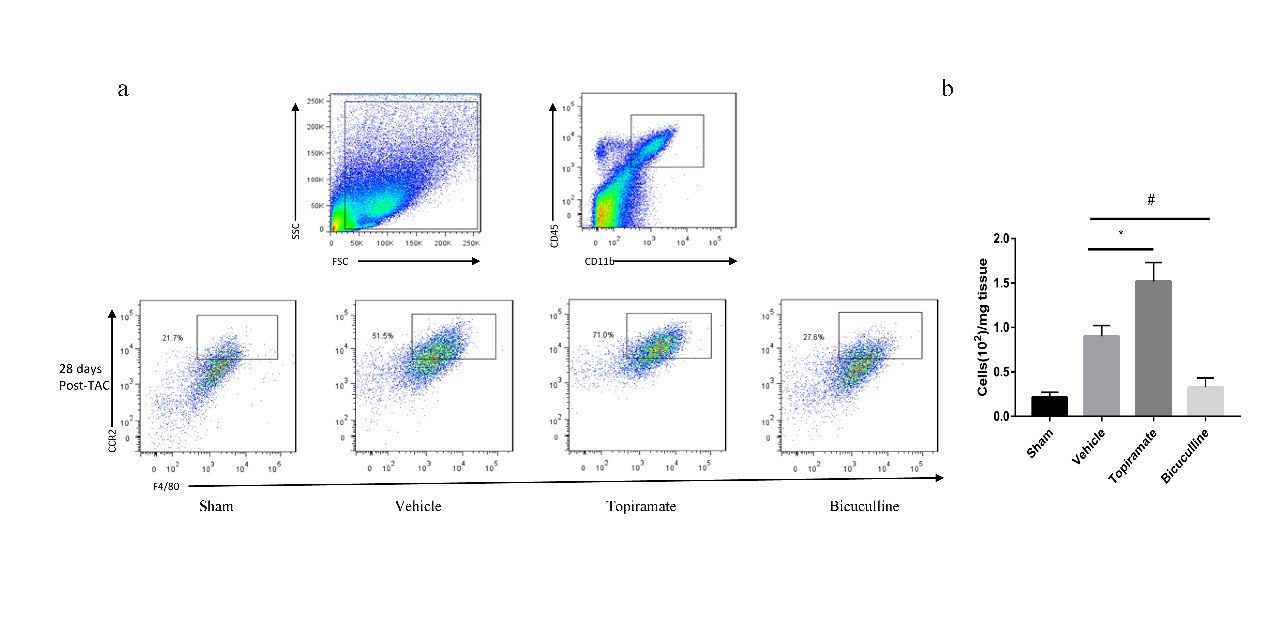


Supplement 7: a. Representative images of CCR2^+^ cardiac macrophage at day 28 post-TAC. b. The number of CD45^+^CD11b^+^F4/80^+^CCR2^+^cell (per mg heart tissue) among the total numbers of live cells isolated from hearts at day 28 post-TAC. For topiramate treatment, *P＜0.05 vs. vehicle. For bicuculline treatment, ^#^P＜0.05 vs. vehicle.


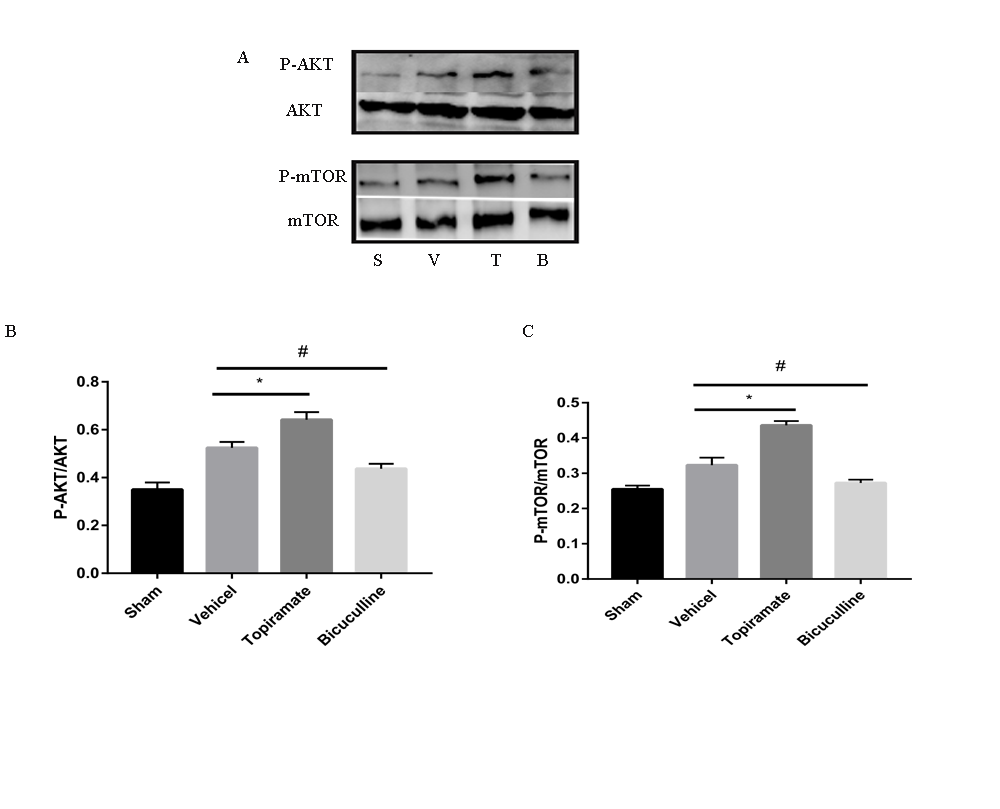


Supplement 8: (a) Representative images of P-Akt and P-mTOR protein at day 7 post-TAC in heart. S: Sham; C: Control; T:Topiramate; B:Bicuculline. (b and c) Quantitative analysis of P-Akt protein (b), and P-mTOR protein (c) at day 7 post-TAC in heart. Data are mean ± SEM, by one-way ANOVA with Bonferroni’s multiple comparison test. For topiramate treatment, *P＜0.05 vs. vehicle. For bicuculline treatment, ^#^P＜0.05 vs. vehicle. (each group, n=6).
